# Financial and functional outcomes in diabetes-related foot disease patients undergoing major lower limb amputation: An observational pilot study from LMIC

**DOI:** 10.12669/pjms.42.(11AASC).15595

**Published:** 2026-04

**Authors:** Nadeem Ahmed Siddiqui, Muhammad Anees, Zia Ur Rehman, Fareed Ahmed Shaikh

**Affiliations:** 1 Dr. Nadeem Ahmed Siddiqui, Consultant Vascular Surgeon, Aga Khan University Hospital, Karachi, Pakistan; 2 Dr. Muhammad Anees, Research fellow, Aga Khan University Hospital, Karachi, Pakistan; 3 Dr. Zia Ur Rehman, Consultant Vascular Surgeon, Aga Khan University Hospital, Karachi, Pakistan; 4 Dr. Fareed Ahmed Shaikh, Consultant Vascular Surgeon, Aga Khan University Hospital, Karachi, Pakistan

**Keywords:** Diabetic nephropathy & vascular disease, Diabetes-related foot disease, Functional outcome, Financial outcome, Major lower limb amputation

## Abstract

**Background and Objectives:**

Major lower limb amputation poses significant financial and emotional challenges especially in diabetic patients. This study aimed to assess the financial and functional outcomes of patients with Diabetic foot disease who underwent major lower limb amputations.

**Methodology:**

This prospective cohort study was conducted at a tertiary care hospital in Pakistan, including Diabetic foot disease patients who underwent major lower limb amputations. A validated questionnaire assessing functional and financial outcomes was administered via telephone six months post-surgery.

**Results:**

The mean age of 41 patients was 54.8±14.6 years with 31 (75.6%) being male. Sepsis was the primary indication for major lower limb amputations (n=15/41, 36.6%). Following MLLA, 50% (n=13/26) of the patients lost their jobs, and only 15.4% (n=4/26) received paid leave. The median cost of prosthetics/crutches (PKR 154,000) far exceeded the average monthly income of patients. Only 17.1% (n=7/41) of patients could afford a nurse/attendant, while 12.2% (n=5/41) had family members who quit their jobs to provide care. Major lower limb amputations significantly reduced overall patient functionality, including ambulation (*p*<0.001) and social functioning (*p*<0.001), with 20% (n=6/30) reporting a decline in physical, and 23% (n=7/30) in emotional aspects of their relationships. Income (*p*=0.477), mode of admission (*p*=0.335), and amputation level (*p*=0.477) did not significantly impact overall functionality post-surgery.

**Conclusions:**

Major lower limb amputations due to diabetic foot disease places significant financial and functional burdens on patients and their families in Pakistan.

## INTRODUCTION

Diabetes mellitus poses a significant public health burden globally. According to the International Diabetes Federation (IDF) Diabetes Atlas (2021), approximately 10.5% of the adult population aged 20-79 years was affected by diabetes. This number is projected to rise to 783 million by 2045, indicating a significant 46% increase.^1^ The prevalence of diabetes in Pakistan, as per the 2022 results of IDF was 26.7%.^2^

Individuals with diabetes mellitus are at an increased risk of severe complications that decrease life expectancy, the overall quality of life, and increase medical care expenses.^3^,^4^ Diabetes-related foot disease (DFD) is a major complication and mainly occurs due to ischemia and neuropathy.^5^ It is seen in 6.3% of individuals worldwide diagnosed with diabetes mellitus.^6^ Patients with DFD are at a significant risk of amputation, with a 10-20 times higher likelihood compared to individuals without diabetes.^7^ Additionally, DFD is linked to a higher risk of mortality when compared to diabetic patients without foot complications.^8^ The incidence of diabetes-related major lower limb amputations (MLLA) is reported to be 94.82 cases per 100,000 individuals diagnosed with diabetes.^9^

The consequences of MLLA go beyond limitations in physical function, demonstrably impacting patients’ social and economic well-being.^10^ In 2001, U.S. healthcare payers incurred approximately $11 billion in costs due to DFD and amputations.^11^ This indicates that lower limb amputations resulting from DFD impose a significant economic burden. Besides the costs incurred during the hospital stay for amputation, MLLA is also associated with indirect expenses, including costs of prostheses, physical therapy, home and vehicle adjustments, and caregiver assistance, all in addition to the considerable psychological consequences of limb loss.^12^ These factors can result in financial challenges and unfavorable health outcomes, including deconditioning, increased risk of higher-level amputation, disease morbidity, and even mortality.^12^ In a retrospective study carried out in Faisalabad, the average direct cost of MLLA was PKR 53,720 ± 12,401($437 ± $101).^13^ While previous studies have evaluated the direct costs of MLLA in our population, there is a lack of research on indirect medical expenses associated with this procedure. Furthermore, given the evolving financial challenges, it is essential to reevaluate the costs related to MLLA in DFD patients. Apart from physical implications, amputation exerts adverse social effects on patients after surgery including social embarrassment and a sense of social isolation.^14^ Although internationally, prior studies have evaluated functional outcomes after MLLA, to the best of our knowledge, no studies have been conducted in our specific setting, which are different in terms of healthcare systems, cultural, religious and societal norms and the financial provisions to address the diseases.^15^,^16^

This study employs a previously content-validated questionnaire from our country to evaluate the financial and functional outcomes following MLLA in patients with DFD.^17^ To the best of our knowledge, this is the first study in Pakistan, assessing indirect costs and functional outcomes after MLLA in DFD patients. The relevance of the previously validated questionnaire is particularly significant in our social and financial context, where the economic conditions are challenging. This pilot study aims to determine financial and functional outcomes after MLLA in patients with DFD.

## METHODOLOGY

This prospective cohort study was conducted in vascular surgery section at The Aga Khan University Hospital. Patients were identified using ICD coding. Study participants were selected via nonprobability consecutive sampling. Medical records of patients were reviewed to gather procedural information. Patients were then contacted via telephone calls six months post-surgery and verbal informed consent (in English or Urdu) was obtained before patient enrollment in the study. In case of any ambiguity or uncertainty, patients were provided with the contact details of the primary research team for further assistance. Patient participation in the study was voluntary, and no financial compensation was offered. The data collected was rendered non-identifiable before entering it into the Excel file to maintain patient confidentiality.

### Ethical Approval:

It was obtained from the Ethics Review Committee (2022-7192-22309; dated: September 10, 2022).

### Inclusion Criteria:

We included all patients above the age of 18 years who underwent below, through- and above-knee amputations due to DFD at the Aga Khan University Hospital from 1st January 2020 to 30th June 2021. Our study excluded patients who did not provide informed consent, underwent amputation for reasons unrelated to DFD (e.g., trauma, tumours), underwent minor amputation, or were unable to participate in telephone interviews.

All phone calls were conducted by a trained team member who underwent standardized training to ask questions using the validated tool in Urdu or English depending on the participant’s preference. The primary research team was involved in providing this training. To ensure sufficient proficiency, two team members independently assessed the interviewer’s clarity in asking questions and their ability to maintain a standardized approach. No member of the research team received any financial compensation.

The questionnaire used in this study is derived from our previous study on content validation of financial and functional outcomes tool in DFD patients undergoing MLLA.^17^ The questionnaire has three sections: demographic data, functional outcomes, and financial outcomes. The functional outcomes section includes the overall ability to perform daily activities, including ambulation and social interaction while the financial impact assesses the financial burden on the patient and their family members post-procedure.

### Statistical analysis:

Data was analyzed via SPSS version 28. Qualitative data like sex, mode of admission, type of amputation, ASA level, etc. were presented as frequency and percentages. Continuous variables like age, inpatient stay (in number of days), number of individuals earning at home, change in household income per month due to surgery, etc. were presented as mean/ median and standard deviation/interquartile range. McNemar’s test was used to analyze the difference between functional outcomes including the ability to walk with support independently and the ability to carry out daily activities. The functionality of patients was evaluated using a Likert scale that ranged from One (never) to Five (always). The Wilcoxon single-ranked test was used to analyze any differences between the two groups and the Hodges-Lehman median difference was used to calculate the effect size. To check for association between financial and functional outcomes, household income per month before the surgery was divided into two categories including per month household income of less than Rs. 50,000 and more than Rs. 50,000. The average per-month income in Pakistan in 2021 was reported to be between Rs. 24,028 and Rs.57,408.^18^ Therefore, the threshold of Rs. 50,000 was chosen for this categorization. The functional outcomes, evaluated using a Likert scale, were combined by summing the scores for each category post-surgery to obtain a total score. An independent sample t-test was used to identify any differences in functional outcomes between the two groups of monthly income. A p-value of less than 0.05 was considered significant.

## RESULTS

A total of 88 patients with DFD underwent major lower limb amputation from 1st January 2020 to 30th June 2021 in our tertiary care hospital. Out of a total of 88 patients, 49 were excluded as they did not meet the inclusion criteria. The reasons for exclusion varied: 23 participants did not respond, 17 were deceased, five were under 18 years of age, and four declined to participate. Therefore, a total of 41 patients were included in the study for final analysis.

The mean age of 41 patients in the study was 54.8 ± 14.6 years with 31 (75.6%) being male and 10 (24.4 %) females. A total of 25 (61%) patients underwent elective MLLA compared to 16 (39%) who underwent emergency MLLA. Patients had multiple complaints upon presentation, with the most prevalent being a discharging wound (n=25, 46.3%) followed by sepsis (n=12, 22.2%). The mean inpatient hospital stay was 7.4 ± 6.3 days. Life-threatening sepsis (n=15, 36.6%) was the primary cause of MLLA, followed by advanced ischemia (n=14, 34.1%) and non-salvageable soft tissue loss (n=12, 29.3%). Below-knee amputation (n=27, 65.9%) was the most common procedure, with above-knee (n=12, 29.3%) and through-knee amputation (n=2, 4.9%) less frequent, (Table-I).

**Table 1:** Demographics of the study participants.

Variable	Frequency (%)
Age(y), mean (SD)	54.8 (14.6)
***Sex:***	
Male	31 (75.6)
Female	10 (24.4)
***Mode of Admission:***	
Elective	25 (61.0)
Emergency	16 (39.0)
***Presenting Complaint: (n=54)***	
Cold feet	2 (3.7)
Discharging wound	25 (46.3)
Sepsis	12 (22.2)
Severe pain	5 (9.3)
Skin discoloration	3 (5.6)
Others	7 (13.0)
***Time difference between symptoms and hospital presentation:***	
< 3 days	5 (12.2)
3-7 days	4 (9.8)
More than a week	32 (78.0)
Previous surgery on the same limb	18 (43.9)
***ASA level:***	
I	2 (4.9)
II	12 (29.3)
III	22 (53.7)
IV	5 (12.2)
***Anesthesia:***	
General	23 (56.1)
Regional / block	3 (7.3)
Spinal	15 (36.6)
Inpatient stay, mean (SD)	7.4 (6.3)
***Reason for amputation:***	
Life-threatening sepsis	15 (36.6)
Non-salvageable soft tissue loss	12 (29.3)
Advanced irreversible ischemia	14 (34.1)
***Level of amputation:***	
Above Knee Amputation	12 (29.3)
Below Knee Amputation	27 (65.9)
Through Knee Amputation	2 (4.9)

**Fig.1:**
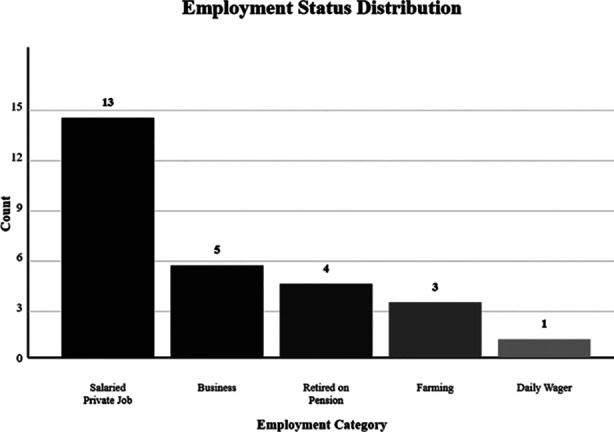
Patient’s occupation before the surgery.

**Fig.2:**
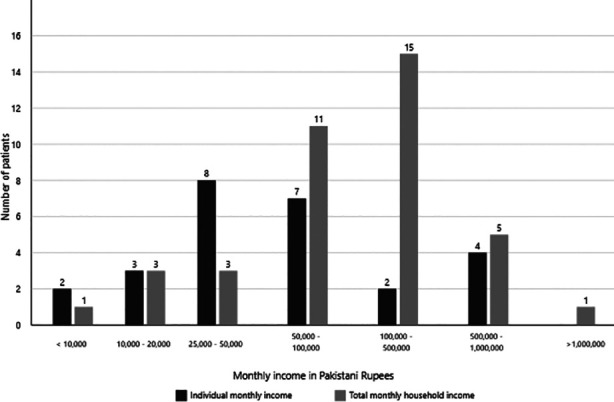
Individual and household monthly income before the surgery in Pakistani Rupees.

In Table-II shows the financial outcomes of the DFD patients who underwent MLLA. A total of 26 (63.4%) patients were employed before the surgery, with 13 (50%) patients having a salaried private job (Fig.1). Most patients (n=8, 30.8%) reported an individual monthly income between Rs 25,000 – 50,000, whereas the total household income for most patients (n=15, 38.5%) ranged between Rs 100,000 – 500,000 (Figure 2). The median number of individuals earning at home was two. Out of the total number of individuals who were employed, 13 (50%) lost their jobs because of the surgery and only four (15.4%) individuals were given paid leave during the time of their surgery. A total of five (12.2%) patients’ family members lost or quit their jobs to help them with their nursing needs. A total of seven (17.1%) patients required re-admission because of complications within a month, the most common being a surgical site infection. The median expenditure on crutches or prosthetic limbs was PKR 154,000 (5,000 – 600,000). Only seven (17.1%) patients were able to afford a nurse or an attendant for their help, with four (57.1%) of them spending less than Rs 30,000 per month. A total of 17 (41.5%) patients received some financial help for their surgery including help from donors (n=2, 4.9%) and hospital welfare (n=11, 26.8%).

**Table-II:** Financial outcomes of DFD patients undergoing MLLA

Financial Outcome	Frequency (%)
Number of individuals employed before the surgery (n=41)	26 (63.4)
Number of employed individuals per household, median (IQR) (n=41)	2.0 (2.0)
Lost their job after the surgery (n=26)	13 (50.0)
Paid leaves during recovery (n=26)	4 (15.4)
Family member quitting job to aid in patient recovery (n=41)	5 (12.2)
***Monthly individual income before the surgery (n=26)***	
< 50,000	13 (50.0)
> 50,000	13 (50.0)
Hospital readmission due to complications (n=41)	7 (17.1)
Cost of prosthetic limb/crutches, median (IQR) (n=26)	72,500 (171,250)
Hired a nurse/attendant (n=41)	7 (17.1)
***Duration of nurse’s/attendant’s assistance (n=7):***	
10 - 30 days	1 (2.4)
1 - 3 months	1 (2.4)
> 3 months	5 (12.2)
***Monthly expense on nurse/attendant (n=7):***	
< 30,000	4 (57.1)
> 30,000	3 (42.9)
Stopped children from going to school/college (n=41)	3 (7.3)
Sold valuables/assets to cover up for the financial loss (n=41)	15 (36.6)
Approximate cost of the valuable/asset, median (IQR), (n=15)	400,000 (2,167,500)
***Received financial help (n=41):***	
None	24 (58.5)
Donors	2 (4.9)
Hospital Welfare	11 (26.8)
Others	4 (9.8)
Change in per month household income due to surgery, median (IQR), (n=27)	125,000 (757,500)

Total of 34 (82.9%) patients were reported to have recovered completely by the time of the interview, six months after the surgery. Patients who were retired and receiving a pension (four out of 26 employed) were included in the category of patients who were employed before the surgery (Table 3). Among the 26 patients who were employed, 13 (50.0%) stopped working post-surgery and identified physical limitation/weakness (n=8, 61.5%) as the primary reason for their inability to continue working. Out of all the patients who continued working post-surgery, five (38.5%) had to change their occupation. Only 30 patients responded to the question on the effect of the surgery on spousal relationships after the surgery. A total of six (20%) patients reported their physical aspect of the relationship with their spouse being affected by the surgery. Seven (23%) patients reported an effect on the emotional aspect of the relationship with their spouse (Table-III).

**Table-III:** Functional outcomes of patients

Functional Outcome	Frequency (%)		
Recovered completely (n=41)	34 (82.9)		
Social relationship affected (n=41)	20 (48.8)		
Regular evaluation of other foot (n=41)	21 (51.2)		
Stopped working after the procedure (n=26)	13 (50)		
***Reason for not working again (n=13):***			
Physical limitation/weakness	8 (61.5)		
Unable to find a similar job.	2 (15.4)		
Others (fired, not able to manage their job, psychological barrier)	3 (23.1)		
Change in occupation (n=13)	5 (38.5)		
Affected physical relationship with the spouse (n=30)	6 (20.0)		
Affected emotional relationship with the spouse (n=30)	7 (23.0)		
	*Pre-procedure*	*Post-procedure*	*p-value*
Ability to walk with support independently (n=41)	40 (97.6%)	36 (87.8%)	0.125
Ability to carry out daily activities (n=41)	38 (92.7%)	29 (70.7%)	0.004

There was no statistically significant difference (*p* = 0.125) between the ability of the patients to walk with support (crutches, pre-procedure, and crutches or prosthesis, post-procedure) independently. However, there was a statistically significant difference (*p* = 0.004) in the ability of patients to carry out daily activities before and after the procedure.

The results of the functionality of the patients before and after the procedure are shown in Table-IV. The results indicate that there was a significant difference (*p* < 0.001) between the functionality of patients, with patients exhibiting poor functionality after the surgery. Patients demonstrated the greatest difference in their ability to do grocery shopping (Hodges-Lehman median difference of -3.00) whereas their ability to walk inside the house showed the smallest difference (Hodges-Lehman median difference of -1.00).

**Table-IV:** Difference in ambulation and social functioning pre- and post-procedure.

Functional outcome category	Functional outcome	Median [IQR] Pre-procedure	Median [IQR] Post-procedure	*p-*value	Hodges-Lehman Median Difference	95% Confidence Interval
Ambulation	Independently able to walk inside the house	5.0 [5.0-5.0]	3.0 [1.0-4.0]	<0.001	-2.00	-2.50, -1.50
	Independently able to walk outside the house	5.0 [5.0-5.0]	3.0 [1.0-4.0]	<0.001	-2.00	-2.50, -1.50
	Independently able to climb stairs	5.0 [5.0-5.0]	2.0 [1.0-3.0]	<0.001	-2.50	-3.00, -2.00
Social functioning	Independently able to attend social gatherings	5.0 [5.0-5.0]	1.0 [1.0-3.0]	<0.001	-2.50	-3.00, -2.00
	Independently able to attend weekly prayers	5.0 [5.0-5.0]	1.0 [1.0-3.5]	<0.001	-2.00	-3.00, -2.00
	Independently able to use transport	5.0 [5.0-5.0]	1.0 [1.0-3.0]	<0.001	-2.50	-3.00, -2.00
	Independently able to do grocery shopping	5.0 [4.0-5.0]	1.0 [1.0-1.0]	<0.001	-3.00	-3.50, -2.00
	Involved in any recreational activity	5.0 [4.0-5.0]	1.0 [1.0-2.5]	<0.001	-2.50	-3.00, -2.00

Patients with incomes both below and above Rs. 50,000 showed similar levels of post-procedure functionality, with no statistically significant difference (*p* = 0.477). There was no statistically significant difference in post-procedure functionality in patients who underwent above, below, and through knee amputation (*p* = 0.477). Mode of admission showed no significant difference in post-procedure functionality as well (*p* = 0.335).

## DISCUSSION

This study assessed the financial and functional impact of MLLA on patients with DFD. This surgical intervention resulted in a financial burden not only for the patients undergoing the procedure but also for their family members. Additionally, the intervention had a significant impact on the functional and social capabilities of the patients. The study found no significant association between individual monthly income and overall functionality of the patients.

Multiple studies in the past have focused on the return-to-work rate after lower limb amputations.^19^-^22^ The documented return to work rates following lower limb amputation (including major and minor amputations) ranged from 43% to 70%.^22^,^23^ However, Wan Hazmy et al. focused specifically on MLLA and reported that 50% of the participants could not continue their pre-amputation jobs.^21^ Our study showed a similar trend as 50% (13/26) of the patients experienced job loss as a direct consequence of undergoing MLLA. The most frequently cited reasons for not being able to work again by Fischer et al included ongoing medical conditions and difficulties associated with amputation or prosthesis use.^24^ However, patients in our study reported physical limitations/weakness and the inability to find a similar job as the primary reasons for not being able to return to work again. The discrepancy observed in the two studies may be attributable to distinct target populations. Our study exclusively focused on patients with DFD who had undergone MLLA whereas Fisher et al. investigated a broader population including all lower limb amputations, regardless of the cause. Additionally, only 15.4% (4/26) received a paid leave of absence during the recovery period. This, coupled with the direct expenses associated with MLLA, imposes a significant financial burden on individuals undergoing MLLA. The added financial burden further contributes to the decreased quality of life.

Our study further evaluated the indirect costs associated with MLLA, including expenses incurred for assistive devices such as crutches and prostheses, as well as home care provided by nurses and attendants. Following MLLA, patients require comprehensive support for their physical, psychological, and social needs.^25^ This often necessitates the assistance of at-home nurses or attendants who can provide essential support in improving the quality of life during the recovery process.^26^ In our study, only 17.1% (7/41) of the patients were able to afford a nurse or an attendant and 12.2% (5/41) of the patients could afford them for over three months. Additionally, the cost of prostheses or crutches was Rs. 72,500 (171,250), which was at least 1.5 times more than the individual monthly income of 50% (13/26) of the patients employed before the surgery (< 50,000). This financial disparity also presents a significant barrier to accessing mobility devices and can potentially lead to poor functional outcomes post-amputation.

Studies in the past have focused on caregiver burden using the Zarit Burden Interview (ZBI-12), Burden Assessment Scale (BAS), and Self-Assessment caregiver questionnaire (CSAQ) scale.^27^,^28^ The ZBI-12, a tool used for assessing caregiver burden in chronic diseases, revealed significantly high scores among caregivers of patients who underwent MLLA.^27^,^29^ Additionally, CSAQ and BAS revealed increased levels of stress and burden on caregivers, respectively.^28^ Despite past studies on caregiver burden, the specific issue of the financial burden faced by caregivers remains unexplored.^27^-^30^ Additionally, the BAS only contains a single item on caregiver financial burden, limiting the depth to which this issue has been explored. This is particularly relevant in our context, where 40% of the population lives below the poverty line.^31^ In our study, 12.2% (5/41) of patients’ family members were forced to quit their jobs to aid in patient recovery imposing a financial burden on the family members. Additionally, 7.3% (3/41) of families experienced disruptions in their children’s education, as they had to stop their children from going to school to assist with patient care and address financial challenges. Furthermore, patients had to desperately sell their valuables and receive financial help from hospital welfare, donors, and others. This underscores the extensive burden placed on families beyond the direct medical costs of MLLA which has far-reaching consequences on the quality of life of patients’ family members.

The decline in ambulation post-MLLA is well documented in the literature.^32^,^33^ According to Chopra et. al less than 50% of patients receiving MLLAs are ambulatory after amputation.^32^ A study by MacCallum et al. showed a decline in ambulation among above-knee amputation (AKA) and below-knee amputation (BKA) patients, with pre-operative ambulation rates of 83.1% and 44.9%, respectively, decreasing to 58.0% and 25.2% post-operatively. Similarly, our study observed a significant decrease in patient ambulation following surgery compared to pre-operative levels further solidifying the previous findings. Postoperative nonambulatory patients have a lower one year estimated survival rate compared to ambulatory patients.^32^ This underscores the importance of prioritizing early post-operative rehabilitation which has been shown to improve survival.^34^ Therefore, comprehensive patient counselling regarding post-operative rehabilitation and ambulatory outcomes should be done to enhance informed decision-making and patient autonomy.

Patients in our study demonstrated a significant decline in all aspects of their social functioning after the surgery, decreasing their overall quality of life. Patients also reported that MLLA adversely impacted their relationships with their spouses. Past studies have focused on SF-36 and EQ-5D-5L to assess the quality of life in patients after MLLA, demonstrating poor quality of life.^32^ A systematic review by Hawkins et. al concluded that there was heterogeneity in measuring the quality of life and functional outcomes post-MLLA, making it difficult to compare studies on MLLA.^35^ However, the questionnaire we used was content validated on our population to assess the financial and functional outcomes making it useful in our context.

### Limitations:

Firstly, its pilot nature led to a limited sample size, hindering the detection of potential associations between monthly income and functional outcomes. Notably, the study observed poor functional outcomes in most patients, likely masking any underlying relationship. Therefore, a larger prospective study with a more robust sample size is necessary to definitively test this association. Secondly, recall bias presents a potential concern, as patients were contacted via phone calls to report their pre-operative functional status. This may introduce inaccuracies due to memory limitations. To address this recall bias, we have included only those MLLA patients who had their procedures done within 18 months of interview phone calls. Lastly, cultural factors within Pakistan may have led to some patients underreporting or not reporting their true monthly income, potentially biasing income-related findings.

The study addresses a significant gap in the existing literature, particularly in the context of Pakistan, by specifically focusing on patients with DFD who undergo MLLA. Moreover, to the best of our knowledge, this is one of the first studies from this region that has comprehensively evaluated the multiple aspects of a financial burden on patients and their caregivers which is particularly relevant in a country where a significant portion of the population lives below the poverty line. The prospective nature of the study allows for temporal association between MLLA and financial and functional outcomes further adding to the strength of the study.

## CONCLUSION

MLLA had a significant and multifaceted impact on patients, including financial constraints, functional limitations, social repercussions, and caregiver burden. This study has identified the substantial financial burden borne by patients and families, including job loss, high prosthesis costs, and limited access to financial aid. Additionally, it has shown a decline in social functioning and quality of life post-operatively, highlighting the importance of comprehensive rehabilitation and psychosocial support. Future studies should focus on a bigger sample size to further assess the financial and functional impact of MLLA on patients with DFD.

### Author’s Contribution:

**NAS:** Conceived, designed, analysis, manuscript writing and editing of manuscript and responsible for the accuracy of the study.

**MA:** Data collection, initial draft of manuscript and addressed revisions.

**ZR:** Collected data, intellectual and clinical insight and reviewed final draft.

**FAS:** Helped with the draft review.

All authors have read and approved the final version and are accountable for the integrity of the study.

## Data availability statement:

Data are available on reasonable request.

## References

[1] International Diabetes Federation [Internet] Facts &figures.

[2] Azeem S, Khan U, Liaquat A (2022). The increasing rate of diabetes in Pakistan:A silent killer. Ann Med Surg.

[3] Forbes JM, Cooper ME (2013). Mechanisms of Diabetic Complications. Physiol Rev.

[4] Deshpande AD, Harris-Hayes M, Schootman M (2008). Epidemiology of Diabetes and Diabetes-Related Complications. Phys Ther.

[5] Edmonds M, Manu C, Vas P (2021). The current burden of diabetic foot disease. J Clin Orthop Trauma.

[6] Zhang P, Lu J, Jing Y, Tang S, Zhu D, Bi Y (2017). Global epidemiology of diabetic foot ulceration:a systematic review and meta-analysis. Ann Med.

[7] Moxey PW, Gogalniceanu P, Hinchliffe RJ, Loftus IM, Jones KJ, Thompson MM (2011). Lower extremity amputations--a review of global variability in incidence. Diabet Med J Br Diabet Assoc.

[8] Boyko EJ, Ahroni JH, Smith DG, Davignon D (1996). Increased Mortality Associated with Diabetic Foot Ulcer. Diabet Med.

[9] Ezzatvar Y, García-Hermoso A (2023). Global estimates of diabetes-related amputations incidence in 2010–2020:A systematic review and meta-analysis. Diabetes Res Clin Pract.

[10] Chigblo P, Tidjani IF, Alagnidé E, Lawson E, Madougou S, Agbessi O (2019). Outcomes of lower limb amputees at Cotonou. J Clin Orthop Trauma.

[11] Margolis DJ, Malay DS, Hoffstad OJ, Leonard CE, MaCurdy T, Tan Y (2011). Economic burden of diabetic foot ulcers and amputations. In:Data Points Publication Series.

[12] Cach G, Haffner ZK, Dekker P, Fan KL, Attinger CE, Evans KK (2023). Financial Toxicity of Lower Extremity Amputation:Providing Supportive Services as Part of a Multidisciplinary Care Model. Plast Reconstr Surg.

[13] Labeeq M, Tariq MA, Tung SA, Yar MA, Rehman W, Ehsan SB (2020). The Economic Impact of Lower Extremity Amputations in Diabetics. A Retrospective Study From a Tertiary Care Hospital of Faisalabad, Pakistan. Rochester, NY.

[14] Washington ED, Williams AE (2016). An exploratory phenomenological study exploring the experiences of people with systemic disease who have undergone lower limb amputation and its impact on their psychological well-being. Prosthet Orthot Int.

[15] Nehler MR, Coll JR, Hiatt WR, Regensteiner JG, Schnickel GT, Klenke WA (2003). Functional outcome in a contemporary series of major lower extremity amputations. J Vasc Surg.

[16] Taylor SM, Kalbaugh CA, Blackhurst DW, Hamontree SE, Cull DL, Messich HS (2005). Preoperative clinical factors predict postoperative functional outcomes after major lower limb amputation:an analysis of 553 consecutive patients. J Vasc Surg.

[17] Siddiqui NA, Khaliq MA, Pirzada MA, Rehman Z, Shaikh F, Riaz A (2024). Development and content validation of a financial and functional outcomes tool for diabetes-related foot disease in patients undergoing major lower limb amputation:a prospective observational study from Pakistan. BMJ Open.

[18] Pakistan Average Monthly Wages |Economic Indicators |CEIC [Internet].

[19] Schoppen T, Boonstra A, Groothoff JW, de Vries J, Göeken LNH, Eisma WH (2001). Employment status, job characteristics, and work-related health experience of people with a lower limb amputation in The Netherlands. Arch Phys Med Rehabil.

[20] Darter BJ, Hawley CE, Armstrong AJ, Avellone L, Wehman P (2018). Factors Influencing Functional Outcomes and Return-to-Work After Amputation:A review of the literature. J Occup Rehabil.

[21] Wan Hazmy CH, Chia WYE, Fong TS, Ganendra P (2006). Functional outcome after major lower extremity amputation:a survey on lower extremity amputees. Med J Malaysia.

[22] Burger H, Marincek C (2007). Return to work after lower limb amputation. Disabil Rehabil.

[23] Penn-Barwell JG (2011). Outcomes in lower limb amputation following trauma:a systematic review and meta-analysis. Injury.

[24] Fisher K (1998). Return to work after lower limb amputation. Arch Phys Med Rehabil.

[25] Mohamed Ahmed Elshenawy A, Bakr A, Hamed H (2023). Health Needs of Patients with Lower Limb Amputation in Postoperative Period. Port Said Sci J Nurs.

[26] Hinkle J, Hinkle JL, Cheever K (2014). The 13th edition of Brunner &Suddarth's Textbook of Medical-Surgical Nursing. Lippincott, Williams &Wilkins, Philadelphia, Pa, 2013.

[27] Çamur S, Batıbay SG, Bayram S (2020). Effect of lower extremity amputation on caregiving burden in caregivers of patients with diabetic foot:Prospective cohort study. Int Wound J.

[28] Costa MSA, Machado JC, Pereira MG (2018). Burden changes in caregivers of patients with type 2 diabetes:A longitudinal study. J Adv Nurs.

[29] Bédard M, Molloy DW, Squire L, Dubois S, Lever JA, O'Donnell M (2001). The Zarit Burden Interview:a new short version and screening version. The Gerontologist.

[30] Costa S, Leite Â, Pinheiro M, Pedras S, Pereira MG (2020). Burden and quality of life in caregivers of patients with amputated diabetic foot. PsyCh J.

[31] Poverty in Pakistan up from 4.4pc to 5.4pc:WB [Internet].

[32] Chopra A, Azarbal AF, Jung E, Abraham CZ, Liem TK, Landry GJ (2018). Ambulation and functional outcome after major lower extremity amputation. J Vasc Surg.

[33] MacCallum KP, Yau P, Phair J, Lipsitz EC, Scher LA, Garg K (2021). Ambulatory Status following Major Lower Extremity Amputation. Ann Vasc Surg.

[34] Stineman MG, Kwong PL, Kurichi JE, Prvu-Bettger JA, Vogel WB, Maislin G (2008). The Effectiveness of Inpatient Rehabilitation in the Acute Postoperative Phase of Care After Transtibial or Transfemoral Amputation:Study of an Integrated Health Care Delivery System. Arch Phys Med Rehabil.

[35] Hawkins AT, Henry AJ, Crandell DM, Nguyen LL (2014). A Systematic Review of Functional and Quality of Life Assessment after Major Lower Extremity Amputation. Ann Vasc Surg.

